# Interaction between tacrolimus and calcium channel blockers based on CYP3A5 genotype in Chinese renal transplant recipients

**DOI:** 10.3389/fphar.2024.1458838

**Published:** 2024-08-29

**Authors:** Huiying Zong, Yundi Zhang, Fengxi Liu, Xiaoming Zhang, Yilei Yang, Xiaohong Cao, Yue Li, Anan Li, Penglin Zhou, Rui Gao, Yan Li

**Affiliations:** ^1^ Department of Clinical Pharmacy, Shandong Provincial Qianfoshan Hospital, Shandong University of Traditional Chinese Medicine, Shandong Medicine and Health Key Laboratory of Clinical Pharmacy, Jinan, China; ^2^ Department of Clinical Pharmacy, The First Affiliated Hospital of Shandong First Medical University and Shandong Provincial Qianfoshan Hospital, Shandong Engineering and Technology Research Center for Pediatric Drug Development, Shandong Medicine and Health Key Laboratory of Clinical Pharmacy, Jinan, Shandong, China; ^3^ Urinary surgery, The First Affiliated Hospital of Shandong First Medical University and Shandong Provincial Qianfoshan Hospital, Jinan, China

**Keywords:** renal transplant, tacrolimus, calcium channel blockers, CYP3A5 polymorphism, C_0_/D

## Abstract

**Objective:**

To investigate the effect of calcium channel blockers (CCBs) on tacrolimus blood concentrations in renal transplant recipients with different *CYP3A5* genotypes.

**Methods:**

This retrospective cohort study included renal transplant recipients receiving tacrolimus-based immunosuppressive therapy with or without CCBs in combination. Patients were divided into combination and control groups based on whether or not they were combined with CCBs, and then further analyzed according to the type of CCBs (nifedipine/amlodipine/felodipine). Propensity score matching was conducted for the combination and the control groups using SPSS 22.0 software to reduce the impact of confounding factors. The effect of different CCBs on tacrolimus blood concentrations was evaluated, and subgroup analysis was performed according to the patients’ *CYP3A5* genotypes to explore the role of *CYP3A5* genotypes in drug-drug interactions between tacrolimus and CCBs.

**Results:**

A total of 164 patients combined with CCBs were included in the combination groups. After propensity score matching, 83 patients with nifedipine were matched 1:1 with the control group, 63 patients with felodipine were matched 1:2 with 126 controls, and 18 patients with amlodipine were matched 1:3 with 54 controls. Compared with the controls, the three CCBs increased the dose-adjusted trough concentration (C_0_/D) levels of tacrolimus by 41.61%–45.57% (*P* < 0.001). For both *CYP3A5* expressers (CYP3A5*1*1 or CYP3A5*1*3) and non-expressers (CYP3A5*3*3), there were significant differences in tacrolimus C_0_/D between patients using felodipine/nifedipine and those without CCBs (*P* < 0.001). However, among *CYP3A5* non-expressers, C_0_/D values of tacrolimus were significantly higher in patients combined with amlodipine compared to the controls (*P* = 0.001), while for *CYP3A5* expressers, the difference in tacrolimus C_0_/D values between patients with amlodipine and without was not statistically significant (*P* = 0.065).

**Conclusion:**

CCBs (felodipine/nifedipine/amlodipine) can affect tacrolimus blood concentration levels by inhibiting its metabolism. The *CYP3A5* genotype may play a role in the drug interaction between tacrolimus and amlodipine. Therefore, genetic testing for tacrolimus and therapeutic drug monitoring are needed when renal transplant recipients are concurrently using CCBs.

## 1 Introduction

Tacrolimus, a calcineurin inhibitor (CNI), is commonly used as a first-line immunosuppressive agent for the prophylaxis of organ transplant rejection in clinical practice (Flahault et al., 2017; Viklicky et al., 2020). Because tacrolimus displays a narrow therapeutic window and high pharmacokinetic variability among individuals, therapeutic drug monitoring (TDM) is recommended to guide dosing (Pulk et al., 2015). The main metabolic enzymes of tacrolimus in the liver and small intestine are the cytochrome P450 (CYP450) 3A4 and 3A5 (Cheung et al., 2019). Part of the variability in the pharmacokinetic characteristics of tacrolimus is attributed to the polymorphisms of the *CYP3A4/5* genes (rs776746), which encode the CYP3A4/5 enzymes. The allelic frequency of CYP3A4*1B is less than 1% in the East Asian population, whereas the minor allele frequency (MAF) of CYP3A5*1 can be as high as 30%, (Birdwell et al., 2015; Nair et al., 2015), making it the most important genetic polymorphism for the individualized dosing assessment of tacrolimus. Individuals carrying one or two functional alleles, CYP3A5*1*1 or CYP3A5*1*3, are termed *CYP3A5* expressers, while those carrying two non-functional alleles, CYP3A5*3*3, are termed *CYP3A5* non-expressers (Barbarino et al., 2013). For Asian populations, in *CYP3A5* non-expressers, selective splicing of the CYP3A5 protein leads to enzyme dysfunction, thereby slowing down the metabolism of tacrolimus, ultimately resulting in changes in tacrolimus trough levels and efficacy (Ragia et al., 2016; Gago-Sánchez et al., 2021; Jain et al., 1999).

The prevalence of hypertension in renal transplant recipients ranges from 70% to 85%, exceeding that in the general population, and typically manifests around 1 week post-transplantation (Ducloux et al., 2002; Halimi et al., 2017). Hypertension after renal transplantation can be effectively controlled through the rational use of antihypertensive medications, often resulting in concurrent administration with immunosuppressants. Drug-drug interactions (DDIs) may occur between the antihypertensive agents and tacrolimus, which may prevent the maintenance of blood concentrations and impair the normal functions of tacrolimus. Transplant recipients receiving multiple drug therapies are at a high risk of drug interactions (Hoffmann and Andersson, 1987). CCBs are among the most commonly used antihypertensive drugs in renal transplant recipients, (Sen et al., 2019), such as felodipine, amlodipine, nifedipine, verapamil, and diltiazem. CCBs act as CYP3A inhibitors, which can inhibit the metabolism of tacrolimus, thereby affecting tacrolimus levels (Jasiak and Park, 2016; Rančić et al., 2015). Due to the narrow therapeutic windows of multiple medications and immunosuppressive agents, transplant patients may be particularly susceptible to adverse drug events caused by DDI (Katoh et al., 2000). The interaction between tacrolimus and CCBs varies widely in clinical practice, possibly necessitating dose adjustments of tacrolimus. Currently, there have been limited research on the interaction between CCBs and tacrolimus in renal transplant patients with different *CYP3A5* genotypes. Therefore, the aim of this study is to evaluate the impact of CCBs on tacrolimus blood concentrations in renal transplant patients with different *CYP3A5* genotypes, further guiding dose adjustments when tacrolimus is co-administered with CCBs.

## 2 Methods

### 2.1 Study design

This single-center retrospective cohort study was conducted at The First Affiliated Hospital of Shandong First Medical University and Shandong Provincial Qianfoshan Hospital. Hospitalized patients who underwent renal transplantation for the first time from January 2015 to December 2023 were included. The inclusion criteria were as follows: 1) age ≥18 years; 2) first renal transplantation; 3) receiving a triple immunosuppressive regimen based on tacrolimus (tacrolimus + mycophenolate sodium + glucocorticoids). The exclusion criteria were: (1) multi-organ transplant patients; 2) taking other medications that affect tacrolimus blood concentration (e.g., strong enzyme inhibitors or inducers of cytochrome P450 enzyme including rifampin, phenytoin sodium, carbamazepine, azoles or proprietary Chinese medicine or herbs that affects tacrolimus blood concentration, etc.); 3) severe hepatic dysfunction (e.g., serum alanine aminotransferase levels >3 times the upper normal limit, total bilirubin >2 mg/dL, or known hepatic cirrhosis); or severe gastrointestinal disease (e.g., severe gastric ulcer, gastric perforation, ulcerative colitis, gastric bypass, banding, or gastric sleeve); 4) patients who were pregnant or breastfeeding; 5) patients with significant rejection of transplanted organs or death from other causes within 1–2 months after the operation.

The included renal transplant patients were divided into combination groups (combined with felodipine or amlodipine or nifedipine) and control groups based on whether they were combined with CCBs. Propensity score matching was conducted for the combination and the control groups using SPSS 22.0 software. Gender, age, body mass index (BMI), days post-transplantation, and hematocrit were taken as covariates to reduce the impact of confounding factors on propensity score matching, as they were closely associated with the C_0_/D levels of tacrolimus (Cheng et al., 2021; Kirubakaran et al., 2020; Mo et al., 2020). The selected caliper value was 0.2. Depending on the type of CCB administered in combination, these variables were matched for participants in the combination and control groups at ratios of 1:1, 1:2, and 1:3, respectively, resulting in a new successfully matched dataset. Subgroup analysis was then performed, with each group further subdivided into *CYP3A5* expressers (CYP3A5*1/*1 or CYP3A5*1/*3) and *CYP3A5* non-expressers (CYP3A5*3/*3).

The Ethics Committee of The First Affiliated Hospital of Shandong First Medical University and Shandong Provincial Qianfoshan Hospital approved the research protocol [YXLL-KY-2023 (018)]. The need for informed consent was waived by The First Affiliated Hospital of Shandong First Medical University and Shandong Provincial Qianfoshan Hospital.

### 2.2 Data collection and patient treatment

Demographic and biochemical characteristics of the enrolled patients were collected from the Hospital Information System (HIS), including gender, age, height, weight, post-transplant days, alanine aminotransferase (ALT), aspartate aminotransferase (AST), total bilirubin (TBIL), serum creatinine (Cr), estimated glomerular filtration rate (eGFR), red blood cell count (RBC), albumin (ALB), hemoglobin (HGB), hematocrit (HCT), *CYP3A5* genotypes, tacrolimus trough concentration (C_0_), and dose (D) of tacrolimus. The eGFR was calculated using the CKD-EPI equation based on serum creatinine at the initiation of CCB therapy (Levey and Coresh, 2012). Blood samples were collected from patients in the morning before taking tacrolimus to ensure that the blood concentration was at a trough. Whole blood samples were collected using EDTA anticoagulant tubes to ensure specimen accuracy. Tacrolimus trough levels were determined in our hospital laboratory using chemiluminescence microparticle immunoassay (Abbott i1000). Tacrolimus concentrations typically reached steady state 2–3 days after administration. For the patients combined with CCBs, the tacrolimus concentrations reached steady state after at least 3 days of combination, while for the control patient group, stable tacrolimus trough concentrations were reached after at least 3 days of fixed-dose tacrolimus administration. Therefore, considering the influence of the above factors, steady-state values were taken when collecting the trough concentration data of tacrolimus.

According to the Kidney Disease: Improving Global Outcomes Clinical Practice Guidelines (KDIGO, 2009) (Kidney Disease: Improving Global Outcomes KDIGO Transplant Work Group, 2009), patients receiving post-transplant immunosuppressive therapy are administered intravenous methylprednisolone sodium succinate on the first day after transplantation, with an initial dose of 500 mg/day, gradually tapering down to 40 mg/day over the first week. In the second week, methylprednisolone tablets are taken sequentially at 40 mg/day, gradually decreasing to 16 mg/day as a maintenance dose. Immunosuppressive maintenance therapy includes oral mycophenolate sodium at a dose of 720 mg twice daily. Tacrolimus is administered orally twice daily, with an initial dose ranging from 0.05 to 0.25 mg/kg/day. Tacrolimus dosage is adjusted based on patient usage and clinical conditions. For combination therapy groups, the dosage of nifedipine is 60 mg per day. The dosage of amlodipine is 5 mg once daily. The dosage of felodipine is 5 mg once daily. Healthcare professionals closely monitor the medication administration, and the team ensures high adherence.

### 2.3 Genotyping

The presence of CYP3A5*3 was detected using the TaqMan polymerase chain reaction (PCR) assay (Applied Biosystems, Foster City, CA, United States). Genomic DNA was extracted from blood samples using the TIANamp Blood DNA Kit (DP348; Tiangen Biotech, Beijing, China), following the manufacturer’s instructions. The primers and sequences for CYP3A5*3 are as follows: forward primer (5′-CCT​GCC​TTC​AAT​TTT​CAC​T-3′); reverse primer (5′-GGT​CCA​AAC​AGG​GAA​GAG​GT-3′). To validate the PCR results, Sanger sequencing was performed using the 3730XL Genetic Analyzer (Applied Biosystems, Foster City, CA, United States) to confirm the presence of CYP3A5*3.

### 2.4 Data analysis

Statistical analysis was conducted using propensity score matching, to align the baseline data of renal transplant patients in the combination and control groups, resulting in a successfully matched dataset. The normality of all data was assessed using the Shapiro-Wilk test. Normally distributed continuous data were expressed as mean ± standard deviation (SD), and between-group comparisons were performed using independent-sample two-tailed t-tests. Abnormally distributed continuous data were presented as median and interquartile range (IQR), and between-group comparisons were conducted using non-parametric tests. Categorical data were presented as frequency and percentage, and between-group comparisons were assessed using the chi-square test. A *p*-value of less than 0.05 was considered statistically significant. All analyses were performed using IBM SPSS Statistics software package version 27.0. Graphs were generated using Microsoft Visio (version 2013) and GraphPad Prism (version 9).

## 3 Results

### 3.1 Participants enrollment

A total of 1715 renal transplant patients were screened. Among these, 436 patients were excluded due of immunosuppressive regimens other than tacrolimus, and 214 patients were excluded because of the lack of blood concentration data for tacrolimus. Additionally, patients with missing data, those treated with strong cytochrome enzyme inhibitors or inducers, those with severe liver dysfunction, and those who died within 1 month of renal transplantation were further excluded. Ultimately, 164 patients were included in the combination group, with 83, 63, and 18 patients combining with nifedipine, felodipine and amlodipine, respectively.

**FIGURE 1:**
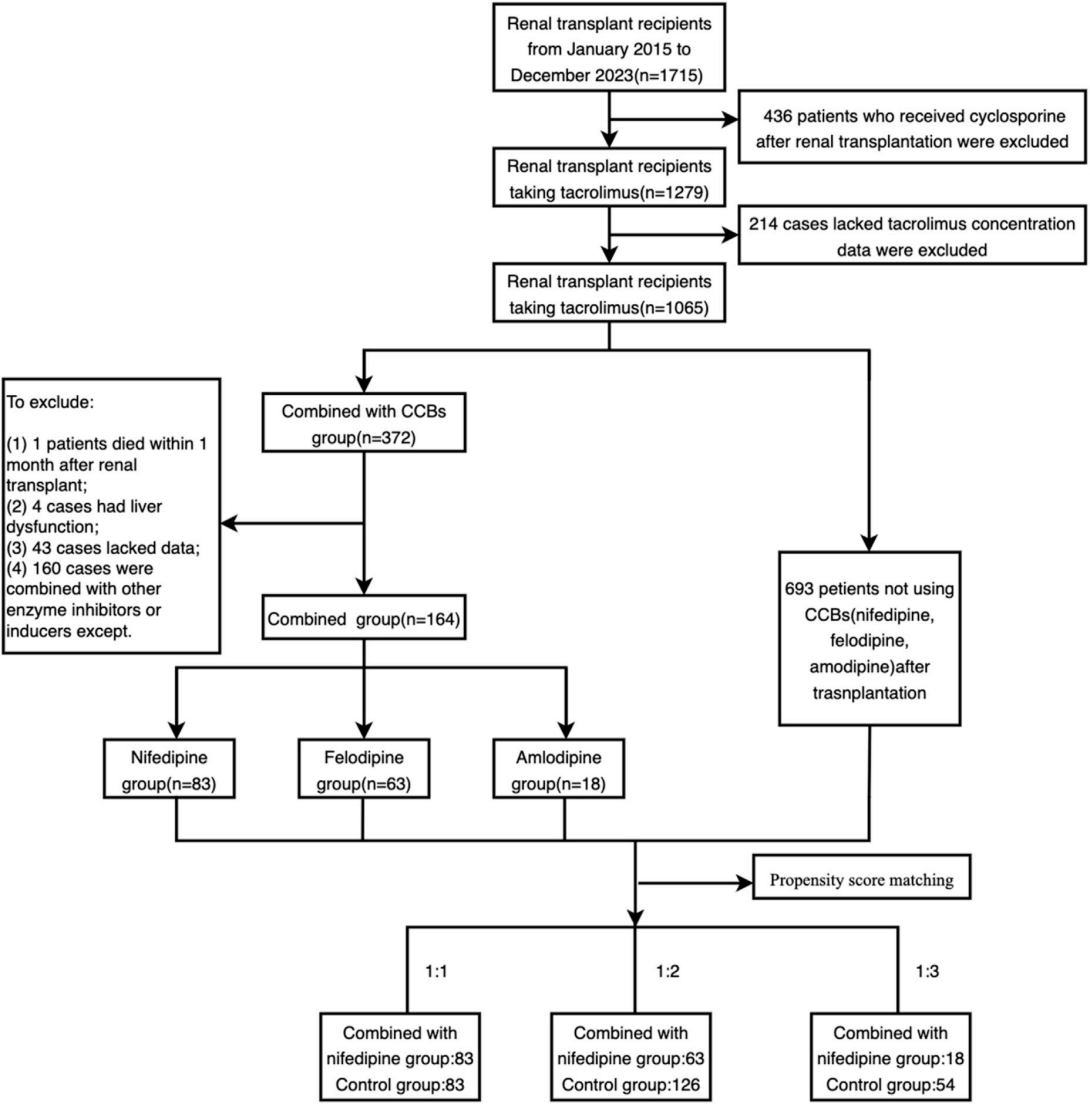
Flow chart of participants in the study.

After propensity score matching, 83 patients with nifedipine were matched 1:1 with the control group, 63 patients who received felodipine were matched 1:2 with 126 controls, and 18 patients with amlodipine were matched 1:3 with 54 controls. The enrollment and propensity matching process are shown in Figure 1. The demographic and baseline characteristics of the participants are presented in Table 1.

**TABLE 1:** Baseline data statistics of combined nifedipine/felodipine/amlodipine and control groups.

	Nifedipine group			Felodipine group			Amlodipine group		
Indicators	Combination (n = 83)	Control-1 (n = 83)	P	Combination (n = 63)	Control-2 (n = 126)	P	Combination (n = 18)	Control-3 (n = 54)	P
Sex of birth (male/female)	64/19	62/21	0.717	45/18	89/37	0.910	13/5	39/15	1.000
Age (years)	43 (35,50)^b^	39 (30,50)^b^	0.143	39 (31,49)^b^	41 (32,50)^b^	0.508	37 (31,44)^b^	36 (31,47)^b^	0.881
BMI (kg/m^2^)	23.53 (20.45,25.88)^b^	22.77 (19.72,25.35)^b^	0.191	22.80 ± 3.74^a^	22.81 ± 3.58^a^	0.986	23.18 ± 3.92^a^	22.24 ± 3.53^a^	0.343
Days post-transplant	9 (7,12)^b^	8 (5,28)^b^	0.898	13 (9,28)^b^	8 (6,51)^b^	0.066	14 (9,53)^b^	31 (6,104)^b^	0.835
Organ dysfunction									
Hepatic dysfunction	0	0	—	0	0	—	0	0	—
eGFR (mL·min^-1^/1.73m^-2^)	52.42 (29.31,82.09)^b^	53.43 (31.32,76.11)^b^	0.778	56.62 (37.57,89.92)^b^	50.62 (27.03,74.58)^b^	0.070	39.85 (20.22,57.96)^b^	54.54 (28.90,75.29)^b^	0.185
HCT (%)	0.33 (0.30,0.36)^b^	0.35 (0.32,0.38)^b^	0.111	0.34 (0.30,0.37)^b^	0.34 (0.30,0.38)^b^	0.400	0.34 ± 0.06^a^	0.33 ± 0.07^a^	0.052
Dose of tacrolimus (mg/kg/d)	0.10 (0.08,0.13)^b^	0.09 (0.07,0.11)^b^	0.062	0.85 ± 0.39^a^	0.88 ± 0.30^a^	0.482	0.83 ± 0.40^a^	0.83 ± 0.31^a^	0.960
CYP3A5 (expressers/non-expressers)	53/30	25/58	—	32/31	41/85	—	8/10	14/40	—

^a^ Mean ± SDs.

^b^ Median and interquartile range, BMI: body mass index, C_0_/D: valley concentration/dosing dose, d: dose of tacrolimus, eGFR: estimate glomerular filtration rate, HGB: hemoglobin, HCT: hematocrit, RBC: red blood cell.

### 3.2 *CYP3A5* genotyping

The genotype frequencies of the CYP3A5*3 polymorphisms in the three groups of patients included in the study are summarized in Table 2. In the nifedipine and Control-1 group, among the 166 renal transplant recipients, 12 recipients (7.22%) carried CYP3A5*1/*1 genotype, 65 (39.16%) carried CYP3A5*1/*3 genotype, and 89 (53.61%) carried the CYP3A5*3/*3 genotype. Consequently, the allelic frequencies of CYP3A5*1 and CYP3A5*3 were 26.80% (89/332) and 73.20% (243/332), respectively. In the felodipine and Control-2 group, among the 189 renal transplant recipients, 11 recipients (5.82%) carried CYP3A5*1/*1 genotype, 62 (32.80%) carried CYP3A5*1/*3 genotype, and 116 (61.38%) carried the CYP3A5*3/*3 genotype. The allelic frequencies of CYP3A5*1 and CYP3A5*3 were 22.22% (84/378) and 77.78% (294/378), respectively. In the amlodipine and Control-3 group, among the 72 renal transplant recipients, two recipients (2.78%) carried the CYP3A5*1/*1 genotype, 28 (38.89%) carried the CYP3A5*1/*3 genotype, and 42 (58.33%) carried the CYP3A5*3/*3. The allelic frequencies of CYP3A5*1 and CYP3A5*3 were 22.22% (32/144) and 77.78% (112/144), respectively. The genotype and allele distributions of *CYP3A5* in all groups were consistent with the Hardy-Weinberg equilibrium (*P* > 0.05 for all groups).

**TABLE 2:** The CYP3A5 genotype distribution of renal transplant patients.

n	Genotype (n/%)			Allele		P
				frequency (%)		
	CYP3A5*1/*1	CYP3A5*1/*3	CYP3A5*3/*3	CYP3A5*1	CYP3A5*3	
N_A_ = 166	12/7.22	65/39.16	89/53.61	26.80	73.20	0.978
N_B_ = 189	11/5.82	62/32.80	116/61.38	22.22	77.78	0.483
N_C_ = 72	2/2.78	28/38.89	42/58.33	22.22	77.78	0.289

N_A_: nifedipine group; N_B_: felodipine group; N_C_: amlodipine group.

### 3.3 The impact of CCBs on tacrolimus concentrations

**FIGURE 2:**
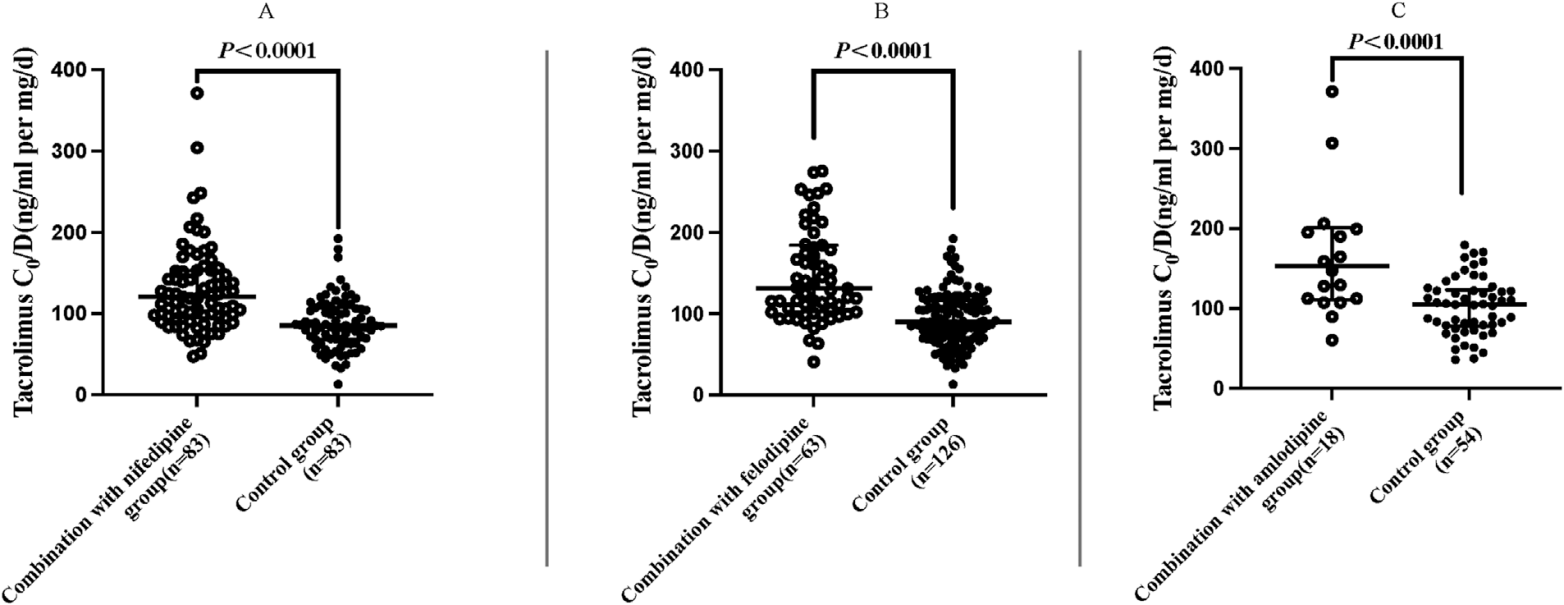
Distribution of C_0_/D values in patients with different CCBs (regardless of genotype). Nifedipine **(A)**, felodipine **(B)** and amlodipine **(C)**. (Hollow circle: C_0_/D levels of tacrolimus combined with CCBs; solid circle: C_0_/D levels of tacrolimus without CCBs).

**FIGURE 3:**
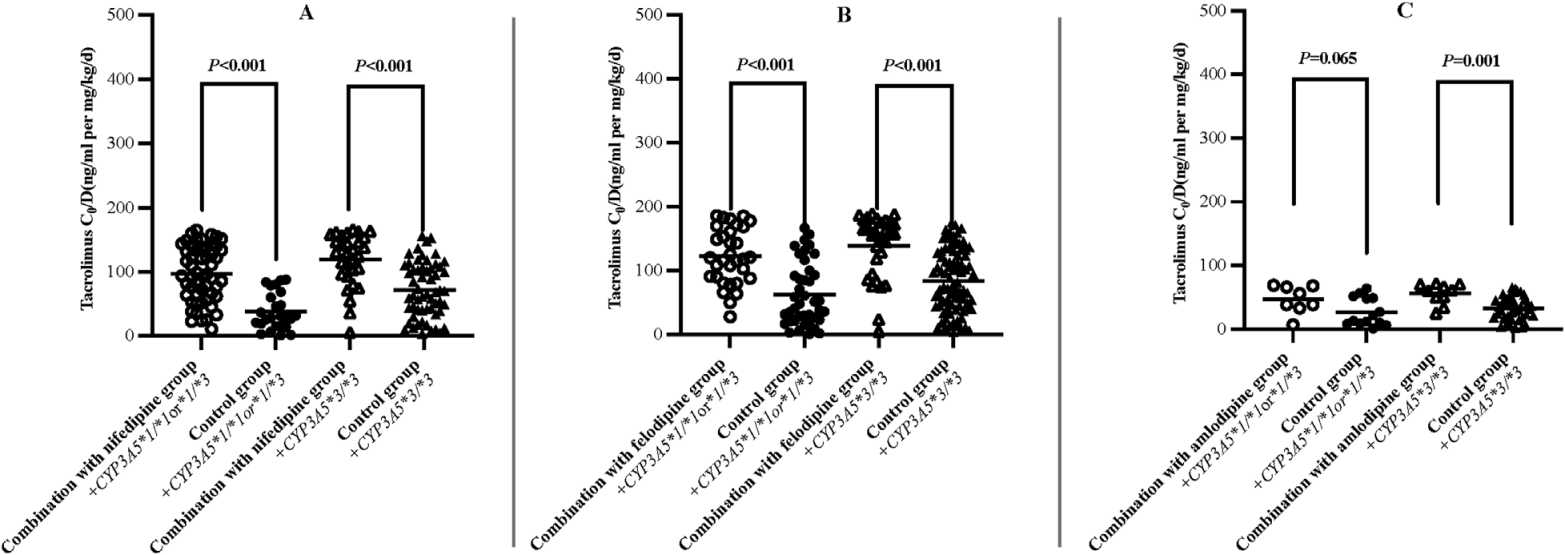
Difference of dose-adjusted trough concentrations of tacrolimus in patients treated with or without nifedipine **(A)**, felodipine **(B)** and amlodipine **(C)** stratified according to CYP3A5 Genotype (Hollow/solid circle: CYP3A5*1/*1 or *1/*3; Hollow/solid triangle: CYP3A5*3/*3).

The results indicated that the C_0_/D values in patients combined with nifedipine [120.61 (94.91, 153.00) ng/mL/(mg/kg/d) vs. 85.17 (68.24, 107.33) ng/mL/(mg/kg/d)], felodipine [131.20 (101.97, 184.28) ng/mL/(mg/kg/d) vs. 90.23 (70.55, 116.78) ng/mL/(mg/kg/d)] and amlodipine [153.14 (111.14,201.20) ng/mL/(mg/kg/d) vs. 105.20 (77.85,123.25) ng/mL/(mg/kg/d) were significantly higher than those in the control groups. Our data indicate that both amlodipine and felodipine are more effective at increasing the blood concentration of tacrolimus compared to nifedipine. Compared with the control groups, the increases in C_0_/D were 45.57% for the amlodipine group, 45.38% for felodipine group, and 41.61% for the nifedipine group (*p* < 0.001 for all groups) (Table 3; Figure 2). For participants combined with felodipine or nifedipine, both CYP3A5*1 and CYP3A5*3 genotype carriers had significantly higher C_0_/D levels than the control groups (*p* < 0.001), as shown in Figures 3A, B. Interestingly, although the tacrolimus C_0_/D levels were significantly higher in all patients combined with amlodipine compared to the control group, when further stratified by genotype, the difference in C_0_/D values between patients with the CYP3A5*1 genotype receiving amlodipine and the control group was not statistically significant (*P* = 0.065). However, for patients with the CYP3A5*3 genotype, the C_0_/D values in those receiving amlodipine were significantly higher than in the control group (*p* = 0.001). The results of the genotype stratification analysis are shown in Figure 3C.

**TABLE 3:** Changes of C_0_/D in patients receiving different CCBs (regardless of genotype).

C_0_/D of tacrolimus (ng/ml per mg/kg/d)				
Type of CCBs combined	Control group	Combination group	%/Different C_0_/D	*P*-Value
Nifedipine	85.17 (68.24,107.33)^a^	120.61 (94.91,153.00)^a^	+41.61	<0.001
Felodipine	90.23 (70.55,116.78)^a^	131.20 (101.97,184.28)^a^	+45.38	<0.001
Amlodipine	105.20 (77.85,123.25)^a^	153.14 (111.14,201.20)^a^	+45.57	<0.001

^a^ median and interquartile range.

## 4 Discussion

In the present study, we collected and analyzed the data from renal transplant patients treated with tacrolimus combined with CCBs, and reported the characteristics of the influence of different CCBs on the blood concentrations of tacrolimus, which provided a valuable reference for the dosage adjustment of tacrolimus in renal transplant recipients. The main findings of the study were as follows: a) CCBs (nifedipine/felodipine/amlodipine) significantly increased the C_0_/D levels of tacrolimus in renal transplant recipients (Table 3; Figure 2). b) For patients receiving felodipine or nifedipine, both *CYP3A5* expressers and non-expressers showed significantly higher C_0_/D values of tacrolimus compared to the controls, regardless of *CYP3A5* genotypes (Figures 3A, B). c) Interestingly, for patients receiving amlodipine, the interaction between the two drugs was influenced by *CYP3A5* genotype: amlodipine had a greater effect on tacrolimus concentration in *CYP3A5* non-expressers than in expressers (Figure 3C).

The inhibition of CCBs on CYP3A leads to the decrease of tacrolimus metabolism and the increase of its blood concentrations (Zhang et al., 2009). CYP3A is an essential enzyme family involved in drug metabolism, metabolizing many drugs and endogenous substances in the human body. The main members of this family are CYP3A4 and CYP3A5. For CYP3A4, CCBs may compete with tacrolimus for the CYP3A4 binding site, reducing the metabolism of tacrolimus. CCB drugs may also alter the conformation of CYP3A4, reducing its catalytic activity on tacrolimus. However, the mutation frequency of CYP3A4 in Asian populations is nearly zero. Therefore, this study does not consider the impact of CYP3A4 mutations on drug interactions. The varying enzyme activity among *CYP3A5* genotype carriers may account for the differences in the extent of this inhibitory effect.

In our previous study, we analyzed the effect of nifedipine on tacrolimus blood concentrations in renal transplant patients with different *CYP3A5* genotypes. It was found that nifedipine significantly increased tacrolimus concentrations in patients with the CYP3A5*3 homozygous genotype, while no significant difference was observed in CYP3A5*1 carriers (Yang et al., 2021). In the present study, we expanded the sample size but found no significant difference in the degree of drug-drug interaction between different *CYP3A5* genotypes. Seifeldin RA et al. have reported DDI between nifedipine and tacrolimus in liver transplant recipients, showing that the daily dose of tacrolimus in the nifedipine group was reduced by 26%, 29% and 38% at 3, 6 and 12 months post-transplantation, respectively (Seifeldin et al., 1997). Butani L et al. found that co-administration of felodipine led to a 3-fold increase in trough concentration levels of tacrolimus in a renal transplant patient (Butani et al., 2002). These findings are broadly consistent with our data. As shown in Table 3, amlodipine and felodipine are more effective in increasing tacrolimus blood concentrations compared to nifedipine. This difference may be related to drug characteristics and other factors. In a prospective study, it was found that the C_0_/D values of tacrolimus were higher in renal transplant recipients co-treated with amlodipine than with nifedipine (157.15 ± 118.11 ng/mL per mg/kg/d vs. 118.76 ± 93.67 ng/mL per mg/kg/d), indicating that different CCBs have different effects on C_0_/D values of tacrolimus (Rančić et al., 2015). Therefore, tacrolimus concentrations need to be monitored in clinical practice to ensure safety and efficacy when combined with CCBs. In addition, Zuo XC et al. found that amlodipine increased the area under the concentration-time curve (AUC) of tacrolimus in *CYP3A5* expressors (48.4 ± 33.8 ngh/mL vs 111.5 ± 109.3 ngh/mL), more significantly than that in non-expressors (129.0 ± 55.7 ngh/mL vs 132.1 ± 50.8 ngh/mL). (Zuo et al., 2013). In our subgroup analysis (Figures 3A,B), the C_0_/D levels in CYP3A5*1 and CYP3A5*3 genotype carriers treated with felodipine or nifedipine were significantly higher than those in the control group. However, the situation was very different with amlodipine. For CYP3A5*1 carriers, the difference of tacrolimus C_0_/D values treated with amlodipine and the control did not reach statistically significance, suggesting that the effect of specific CCBs on tacrolimus metabolism may be related to *CYP3A5* genotypes. Unfortunately, relevant researches are still limited. Further studies are needed to understand better the impact of CCBs on tacrolimus concentrations in patients with different genotypes, which will provide stronger support for individualized drug therapy in clinical practice.

There are still limitations in this study. First, this was a retrospective cohort study with a relative small sample size. We used propensity matching in the stratified analysis to match experimental and control groups by characteristic variables, reducing bias in the treatment selection. However, differences in the duration of tacrolimus treatment may still introduce confounding factors and bias. Second, the study was conducted in a single center, limiting the generalizability of our findings. A large-scale, multi-center study is needed to verify the results better and guide tacrolimus dosage adjustments in renal transplant recipients.

According to our results, the combination of nifedipine or felodipine or amlodipine was associated with a significantly increased blood concentration of tacrolimus in renal transplant recipients (C_0_/D increase by 41%∼46%). Although there was no significant difference in patients with different CYP3A5 genotypes in the cases of nifedipine and felodipine, we observed a difference in tacrolimus concentrations between CYP3A5 expressers and non-expressers with amlodipine. Therefore, it is recommended that therapeutic drug monitoring be conducted in renal transplant recipients when combining CCBs to monitor tacrolimus blood concentrations and appropriately adjust the dose, especially at the initiation of combination therapy, as well as during changes in CCB type, dosage, or discontinuation.

## Data Availability

The raw data supporting the conclusions of this article will be made available by the corresponding author upon reasonable request.

## Abbreviations

ALB, Albumin; ALT, Alanine aminotransferase; AST, Aspartate aminotransferase; AUC, Area under the concentration-time curve; BMI, Body mass index; C_0_, Trough concentration; CCBs, Calcium channel blockers; C_0_/D, Trough concentration/dose; CNI, Calcineurin inhibitors; Cr, Serum creatinine; CYP450, Cytochrome P450; DDIs, Drug-drug interactions; eGFR, Estimated glomerular filtration rate; FPIA, Fluorescence polarization immunoassay; HCT, Hematocrit; HGB, Hemoglobin; HIS, Hospital Information System; IQR, Interquartile range; MAF, Minor allele frequency; RBC, Red blood cell; RSD, Relative standard deviation; RT-PCR, Real-time polymerase chain reaction; TBIL, Total bilirubin; TDM, Therapeutic drug monitoring; SD, Standard deviation.
